# Determinants of adherence to disease modifying anti-rheumatic drugs in White British and South Asian patients with rheumatoid arthritis: a cross sectional study

**DOI:** 10.1186/s12891-015-0831-8

**Published:** 2015-12-29

**Authors:** Kanta Kumar, Karim Raza, Peter Nightingale, Robert Horne, Sarah Chapman, Sheila Greenfield, Paramjit Gill

**Affiliations:** Primary Care Clinical Sciences, University of Birmingham, Birmingham, B15 2TT United Kingdom; Department of Rheumatology, Sandwell and West Birmingham Hospitals NHS Trust, Birmingham, B18 7QH United Kingdom; University of Manchester, Faculty of Medical and Human Sciences, Manchester, M13 9PL United Kingdom; Institute of Inflammation and Aging, College of Medical and Dental Sciences, University of Birmingham, Birmingham, B15 2TT United Kingdom; The Wolfson Building, University Hospitals Birmingham NHS Foundation Trust, Birmingham, B15 2TH United Kingdom; Centre for Behavioural Medicine, Department of Practice and Policy, UCL School of Pharmacy, Mezzanine Floor, Entrance A, BMA House, Tavistock Square, London, WC1H 9JP UK

**Keywords:** Rheumatoid arthritis, DMARDs, Adherence, Beliefs about medicines, Illness representation, Satisfaction with information, Ethnicity

## Abstract

**Background:**

Rheumatoid arthritis (RA) is a common chronic inflammatory disease causing joint damage, disability, and reduced life expectancy. Highly effective drugs are now available for the treatment of RA. However, poor adherence to drug regimens remains a significant barrier to improving clinical outcomes in RA. Poor adherence has been shown to be linked to patients’ beliefs about medicines with a potential impact on adherence. These beliefs are reported to be different between ethnic groups. The purpose of this study was to identify potential determinants of adherence to disease modifying anti-rheumatic drugs (DMARDs) including an assessment of the influence of beliefs about medicines and satisfaction with information provided about DMARDs and compare determinants of adherence between RA patients of White British and South Asian.

**Methods:**

RA patients of either White British (*n* = 91) or South Asian (*n* = 89) origin were recruited from secondary care. Data were collected via questionnaires on patients’: (1) self-reported adherence (Medication Adherence Report Scale-MARS); (2) beliefs about medicines (Beliefs about Medicines Questionnaire-BMQ); (3) illness perceptions (Illness Perceptions Questionnaire-IPQ) and (4) satisfaction with information about DMARDs (Satisfaction with Information about Medicines questionnaire-SIMS). In addition, clinical and demographic data were collected.

**Results:**

The results revealed that socio-demographic factors only explained a small amount of variance in adherence whereas illness representations and treatment beliefs were more substantial in explaining non-adherence to DMARDs. Patients’ self-reported adherence was higher in White British than South Asian patients (median 28 (interquartile range 26–30) vs median 26 (interquartile range 23–30) respectively; *P* = 0.013, Mann–Whitney test). Patients who reported lower adherence were more dissatisfied with the information they had received about their DMARDs (*P* < 0.001, Spearman correlation, SIMS action and usage subscale; *P* < 0.001, Spearman correlation, SIMS potential problems subscale) and had more negative beliefs about their DMARDs and were related to ethnicity with South Asian patients having more negative views about medicines.

**Conclusions:**

Socio-demographic factors were found to explain a small amount of variance in adherence. Illness representations and treatment beliefs were more important in explaining non-adherence to DMARDs. Clinicians managing South Asian patients with RA need to be aware that low adherence may be linked to negative beliefs about medicines and illness representations of RA.

## Background

Rheumatoid arthritis (RA) is a chronic inflammatory disease characterized by an erosive synovitis [1], which can result in joint damage, loss of joint function [2], reduced life expectancy [3] and increased sickness absenteeism [4]. To reduce the risk of joint damage and functional impairment, the National Institute for Health and Clinical Excellence (NICE) [5] has recommended the timely use of disease-modifying anti-rheumatic drugs (DMARDs), ideally in combination and commenced within the first three months of symptom onset [5]. A high degree of adherence by patients to their prescribed regimen is necessary for optimal outcomes [6, 7]. However, poor adherence to drug regimens remains a significant barrier to improving clinical outcomes in RA [8] with only 58–82 % of RA patients adhering to their DMARDs [8]. Non adherence can occur due to practical factors, such as difficulty opening medication packaging and perceptual factors such as doubts about personal need or concerns about potential side effects [9]. Some patients experience inefficacy while others have to stop taking DMARDs because of side effects [10, 11]. Adherence to treatment may be influenced by the patient’s social background, by their beliefs about their illness representations and beliefs about treatments [12].

Evidence suggests that beliefs about medicines that drive non-adherence may be more prevalent in some ethnic groups [13]. Whilst there are recognised differences in beliefs about medicines, the literature describing adherence levels is scarce amongst South Asian RA patients. Our previous research has shown that, South Asian RA patients hold more negative beliefs about DMARDs than White British RA patients [14] and prefer traditional medicines, such as Ayurveda, as opposed to conventional therapy [15, 16]. Moreover, our findings suggested that patients’ evaluations of prescribed DMARDs were influenced by how they judged their personal need for treatment relative to their concerns about potential adverse effects of treatments. We do not, however, know the extent to which these and other beliefs about medicines impact on medication adherence in South Asian patients.

Other studies demonstrating an association between beliefs about medicines and adherence (e.g. in asthma [17], HIV [18] and in RA [19]), have mainly been conducted amongst Hispanic groups or European populations. Furthermore, the literature shows that patients’ views about specific prescribed medicines (necessity beliefs and concerns) are influenced by more general beliefs about pharmaceuticals as a whole and by common-sense understandings of the illness and symptom expectations and experiences [17, 20]. In addition, receiving adequate information about medicines may also be crucial in patients’ decision making [21]. Thus, we wanted to identify potential determinants of adherence to disease modifying anti-rheumatic drugs (DMARDs) including an assessment of the influence of beliefs about medicines and satisfaction with information provided about DMARDs and compare determinants of adherence between RA patients of White British and South Asian [9].

## Methods

This study was conducted in the outpatient Rheumatology departments of Sandwell and West Birmingham Hospitals NHS Trust, Heart of England Foundation NHS Trust and University Hospitals Birmingham NHS Foundation Trust, UK. Consecutive patients with RA were approached during their normal routine rheumatology appointments. To reduce interview bias, the researcher did not approach any patients who were under her care. The researcher approached patients while they were waiting to see their consultant or nurse specialist and discussed details of the study with them. If patients expressed an interest in taking part, they were given a Patient Information Sheet outlining the study and time to ask questions before being asked for their consent. Those patients who required more time to decide were given the researcher's phone number and asked to contact her if/when they decided to participate. In this situation, an additional visit was required on the part of the patients to complete the study forms. Patients were recruited if they: (1) Self-defined themselves as being of either White British or South Asian origin and in addition for ‘South Asian’ origin three or more grandparents born in India or Pakistan; and for ‘White British’ patients had three or more grandparents born in the UK or Ireland as outlined in our published protocol. This approach was used in our previous work [13, 22]. (2) Fulfilled classification criteria for RA [23]. (3) Had been taking at least one DMARD for over 3 months according to their medical records. This cut off was chosen to ensure that participants had a chance to experience taking DMARDs. A number of questionnaires were used to collect data from patients. These were the following;

1. Medication Adherence Report Scale (MARS-6). [24, 25] This self-report measure of adherence has demonstrated good psychometric qualities in a range of illness groups [17, 24] and was used in the present study to assess self-reported adherence to DMARDs. Responses to 6 items assessing the frequency of both unintentional (e.g. ‘*I forgot*’) and intentional (e.g. ‘*I decided to miss a dose*’) non-adherent behaviours were recorded using a 5-point Likert type scale. The score range for the scale is 6 to 30 with higher scores indicating higher reported adherence. Some previous studies have dichotomised the MARS scores [26]. The overall MARS scores were used in the majority of the analyses but patients were also categorized into high or low adherers (MARS ≥ 26 or < =25) (Fig. 1) as stated in our protocol [22]. Adherence to DMARDs was self-reported by patients in this study.

**Fig 1:**
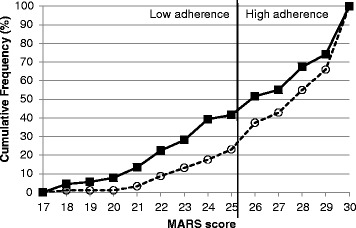
Showing vertical line of the dichotomous MARS. (Square = South Asian patients, circles = White British)
2. The Beliefs about Medicines Questionnaire (BMQ) [27] has Specific and General versions. The BMQ-Specific assesses patients’ beliefs about a particular medicine (in this case RA patients’ belief about DMARDs) and comprises two scales. The 5-item Specific Necessity scale assesses beliefs about personal need for DMARDs (a higher score indicates a stronger belief in personal need for the DMARDs). The Specific Concerns scale comprises 6 items assessing concerns about the potential adverse consequences of taking DMARDs (a higher score indicates stronger concerns about the potential adverse consequences of DMARDs). Adjusted Specific Necessity and Concerns scale scores were calculated by dividing total scores by the number of items in the scale (possible range 1–5). A Necessity-Concerns Differential score (NCD; range −4 to + 4) specific for DMARDs was calculated by subtracting the adjusted Specific Concerns score from the adjusted Specific Necessity score. Patients’ NCD scores are positive if the Necessity beliefs are rated more highly than Concerns and negative if Concerns are rated more highly than Necessity beliefs. The BMQ-General comprises two scales that deal with more general views about medicines as a whole. The 3-item General Overuse scale assesses beliefs about the way in which medicines are used by doctors and the extent to which doctors place too much emphasis on and trust in medicines (higher scores indicate higher agreement with the premise that medicines are overused by doctors). A 5-item General Harm scale assesses beliefs about the intrinsic properties of medicines and the degree to which they are perceived as essentially harmful (with a higher score indicating stronger views about medicines being harmful). Again, adjusted scale scores were calculated by dividing total scores by the number of items in the scale (possible range 1–5).
3. The Satisfaction with Information about Medications (SIMS) [21] (in this case RA patients’ satisfaction with information about DMARDs) comprises 17 items to assess the type of information that patients require in order to facilitate the safe self-management of medication. Each item refers to a particular aspect of their medicines. Examples include “*How to use your medicine*” and “*What you should do if you experience unwanted side effects*”. Patients rated their perception of the quality of information they had received about aspects of their medicine as ‘too much’, ‘none’, ‘too little’ (all scored as 0) or ‘none needed’ or ‘about right’ (scored as 1). The SIMS has two components: (1) ‘Action and usage of medicines’ refers to information received about, for example, how medicines work to control the condition and how to use medicines (nine items; scores range from 0 to 9 with a higher score indicating higher satisfaction) and (2) ‘Potential problems’ refers to information received about, for example, what patients should do if experiencing side effects of the medicine (eight items; scores range from 0 to 8 with a higher score indicating higher satisfaction).
4. The Illness Perceptions Questionnaire (IPQ) [28] measures patients’ perceptions about illness representations (in this case patients’ illness perceptions about RA). Patients’ responses are recorded on a 5–point scale capturing the following dimensions: the chronicity of RA, a cyclical timeline (5 items about the fluctuating nature of RA), the consequences of RA (6 items about the impact of RA), personal control (6 items representing positive beliefs about one’s own ability to control RA), treatment control (5 items representing a belief that treatment is effective), illness coherence (5 items about the patient’s personal understanding of RA), and emotional representation (6 items about emotions caused by RA).
5. The Health Assessment Questionnaire (HAQ) [29] measures patients’ functional status and includes questions related to activities that involve both upper and lower extremities. The HAQ measures the ability to perform 20 activities of daily living with four response categories (without any difficulty (score 0), with some difficulty (score 1), with much difficulty (score 2), not being able to do (score 3)). A higher score indicates a higher level of disability.

In addition, data on socio-economic variables were collected (for example age, gender, occupation and ethnicity) along with clinical data including the disease activity score (DAS). Felson et al. [30] the DAS28 data were extracted from routine follow up clinics around the time when patients took part in the study (within 1–2 months), the justification for their use in this study has been outlined in our study protocol [22].

To accommodate non-English speakers, all questionnaires were translated into Punjabi, Urdu and Hindi using established guidelines [31]. The translations were audio recorded. In addition, English versions of the questionnaires were audio recorded and those who read and spoke English were given a choice of either reading the questionnaires or listening to the audio version. There were 42 patients who requested the audio versions of the questionnaires (30 who required the Punjabi, Urdu or Hindi translation and 12 who were from a White background and requested the English audio tape to be played). Where the audio version was used, responses were indicated by patients and the researcher recorded the patient’s verbal response on the questionnaires. The Cronbach’s alpha between the responses given by patients who filled in the questionnaires themselves and patients who listened to the audio version of the questionnaire and for whom the researcher marked the patients’ responses on the questionnaire were checked for reliability of the responses. There were no inconsistencies between responses. Reliability was adequate for both English and non-English versions.

The Index of Multiple Deprivation (IMD) scores is used widely in England as a measure of deprivation. These scores are derived from patients’ postcodes to measure deprivation, higher scores indicating a higher level of deprivation. They are based on government statistics rating the overall deprivation of small areas (containing approximately 1500 people) by combining a number of deprivation indicators such as local crime, disability, income, education and housing [32].

The study was approved by the South Birmingham Research Ethics Committee and written consent was obtained from all participants.

### Statistical analysis

Data were analysed using PASW Statistics software version 18 (SPSS, Chicago, Illinois). Categorical data are summarised as counts and percentages and other data are presented as means and standard deviation if normally distributed or as medians and interquartile ranges where data were skewed. The *t*-test, Mann–Whitney test, and Fisher’s exact test were used to compare the demographic data and BMQ/SIMS/IPQ. Mann–Whitney and Kruskal Wallis tests were used to compare MARS scores for categorised variables (followed by Dunn’s test where appropriate). Spearman correlations were used to assess the association between continuous variables and MARS scores. A multivariable general linear model was used to predict overall MARS scores. The variables that were adjusted in this model were pre-specified in our study protocol [22]. Linearity was assessed by examination of the relevant boxplots of MARS scores. Binary logistic regression was used to carry out analyses of variables with dichotomous MARS scores. All *P* values are unadjusted.

## Results

### Patient characteristics

Patients were recruited from hospital outpatient clinics. In total, 310 patients were identified and 92 were excluded due to either cancellation of appointments or the patient not attending their appointment. We approached 218 patients of whom 38 (17.4 %) declined to take part in the study, leaving a sample size of 180, a response rate of 58.1 %. There were no differences noted in ethnicity, gender or age between patients who were recruited into the study and those who declined. For logistical reasons including access to space in which to conduct the research the White British group (91 patients) were recruited during the first four months followed by South Asians (89 patients) during the remaining four months. The demographic details of patients are shown in Table 1. South Asian patients were younger (*P* = 0.006, *t*-test) and included a significantly greater proportion of women than the White British group (*P* = 0.024, Fisher’s exact test). There were no significant differences between the two groups in the level of education received (*P* = 0.538, Mann–Whitney). There were differences in employment status between the two ethnic groups (*P* = 0.001, Fisher’s exact test), such that a higher proportion of the South Asian population were homemakers. There were significant differences between patient languages and patients’ self-reported level of education received between the ethnic groups. There were more English speaking patients in the White British group (*P* < 0.001, Fisher’s exact test) and a greater proportion of patients were literate in the White British group. There were also significant differences in the languages that patients spoke with their GPs (*P* < 0.001, Fisher’s exact test).

**Table 1:** Demographic data for all participants. Unless otherwise indicated data are number (%) or median (interquartile range)

	White	South Asian	*P* value
Number	91	89	
Age, years; mean (SD)	57.74 (12.74)	52.46(12.94)	0.006^a^
Female	56 (61)	69 (77)	0.024^b^
Level of education			0.538^c^
Primary	0 (0)	11 (12)	
Secondary	49 (54)	33 (38)	
College	21 (23)	26 (30)	
University	21 (23)	18 (20)	
Number of years of education	14 (11–16)	15 (11–17)	0.439^c^
Employment			0.001^b^
Full time	33 (36)	31 (35)	
Part time	3 (3)	8 (9)	
Unemployment	3 (3)	1 (1)	
Never employed	0 (0)	1 (1)	
Not working due to RA	12 (13)	15 (17)	
Not working for other reason	19 (21)	7 (8)	
Home maker**	4 (4)	18 (20)	
Retired	17 (19)	8 (9)	
Preferred language spoken by patient			<0.001^b^
English	91 (100)	51 (57)	
Punjabi	0 (0)	29 (33)	
Urdu	0 (0)	6 (7)	
Hindi	0 (0)	3 (3)	
Language spoken with GP			<0.001^b^
English	91 (100)	68 (76)	
Punjabi	0 (0)	16 (18)	
Urdu	0 (0)	3 (3)	
Hindi	0 (0)	2 (2)	
Patient Literacy***			<0.001^b^
Yes	90 (99)	71 (80)	
No	1 (1)	18 (20)	
Number of years in UK, mean (SD)	NA	31.76 (11.6)	
DAS CRP, mean (SD)	4.02 (0.83)*	3.76 (0.81)*	0.034^a^
Disease duration (years)	5 (2–11)	7 (3–13)	0.173^c^
Oral/biologic DMARDs			
Methotrexate	77 (84.6)	81 (91.0)	0.256^b^
Sulphasalazine	38 (41.8)	42 (47.2)	0.549^b^
Hydroxychloroquine	10 (11.0)	11 (12.4)	0.820^b^
Anti-TNF	45 (49.5)	35 (39.3)	0.181^b^
Other	8 (8.8)	7 (7.9)	1.000^b^

^a^t-test, ^b^Fisher’s exact test, ^c^Mann-Whitney, *(CRP was available on 91 White British, 86 South Asian patients (Level of education; was available on 91 White British patients, and 88 South Asian patients) **(A homemaker is defined as “a person who manages the household of his or her own family, especially as a principal occupation) ***(patients’ ability to read and write in their preferred language)

### Disease related variables

The DAS28 calculated using the CRP (DAS28-CRP) was higher in the White British patients than the South Asian patients (*P* = 0.034, *t*-test). The disease duration and HAQ did not differ between the two ethnic groups. Furthermore, there were no significant differences between DMARDs that patients were taking amongst the two groups.

### Beliefs about medicines and ethnicity

The Specific Concern (*P* < 0.001, Mann–Whitney), General Overuse (*P* < 0.001, Mann–Whitney), and General Harm (*P* < 0.001, Mann–Whitney) scores were significantly higher in the South Asian patients, indicating more negative views of medicine in general and with regard to their DMARDs in particular (Table 2). There was no significant difference between groups in the Specific Necessity scores for DMARDs. The NCD score was significantly higher in the White British patients (*P* < 0.001, Mann–Whitney) indicating that their beliefs that DMARDs were necessary outweighed their concerns about DMARDs to a greater extent than was seen for the South Asian patients.

**Table 2:** Questionnaire data for all participants. Unless otherwise indicated data are number (%) or median (interquartile range)

Questionnaires			
BMQ			
Specific Necessity	4.00 (3.80–4.20)	4.00 (4.00–4.00)	0.833^c^
Specific Concern	3.50 (2.83–4.00)	4.00 (3.83–4.00)	<0.001^c^
NCD	0.33 (0.00–1.10)	0.00 (0.00–0.17)	<0.001^c^
General Overuse	2.67 (2.00–3.33)	3.33 (3.00–4.00)	<0.001^c^
General Harm	2.40 (2.00–3.00)	3.60 (3.00–4.00)	<0.001^c^
SIMS			
SIMS action and usage	9 (7–9)	8 (6–9)	0.006^c^
SIMS potential problems	6 (5–8)	6 (4–8)	0.060^c^
HAQ	1.25 (1–1.38)	1.25 (1–3)	0.927^c^
IPQ			
Identity	6 (5–8)	6 (5–7)	0.851^c^
Timeline	24 (23–27)	24 (22–25)	<0.001^c^
Consequences	22 (18–24)	22 (19–24)	0.794^c^
Personal control	20 (17–23)	19 (17–23)	0.626^c^
Treatment control	16 (14–18)	16 (15–18)	0.914^c^
Illness coherence	18 (14–20)	15 (11–20)	0.041^c^
Timeline cyclical	15 (14–16)	16 (14–16)	0.017^c^
Emotional representation	22 (18–24)	24 (22–24)	0.004^c^

^c^Mann-Whitney

### Illness perception and ethnicity

There were significant differences between ethnic groups in the IPQ domains. Illness coherence (patients’ understanding of RA) (*P* = 0.041, Mann–Whitney) was lower in the South Asians, indicating that South Asians were more likely to have a poor understanding of RA (Table 2). The timeline (patients’ view of disease as acute/chronic; *P* < 0.001, Mann–Whitney), timeline cyclical (fluctuant disease; *P* = 0.017, Mann–Whitney) and emotional representation (emotions generated in patients; *P* = 0.004, Mann–Whitney) were significantly different between the two groups indicating that South Asians were more likely to view RA as short lived rather than chronic, to experience RA symptoms to be more fluctuating and to feel more negative emotion related to RA.

### Correlations between SIMS, BMQ and IPQ domains within each ethnic group

South Asian patients’ views about medicines being overused and harmful were significantly correlated with levels of satisfaction with information about how medicines work to control RA symptoms and potential issues associated with DMARDs (action and usage (*P* = 0.011) and potential problems (*P* = 0.004). Whereas only the correlations between overuse beliefs and the satisfaction with information about medicines subscales were significant in the White British patients (action and usage (*P* = 0.027); potential problems (*P* = 0.028) but harm scores were not (Table 3). The correlation between patients’ perception of the adequacy of information about their prescribed DMARD and their beliefs about medicines in general were stronger in the South Asian patients. White British patients with higher concerns about the potential adverse effects of their medications had lower SIMS potential problems scores (*P* = 0.031). This was also seen for the White British group with lower necessity-concern differential; (*P* < 0.001).

**Table 3:** Correlation between SIMS and BMQ within each ethnic group

	White British	*P* value	South Asian	*P* value
Action and usage				
Necessity	0.135	0.201	0.019	0.863
Concern	−0.103	0.330	−0.049	0.650
NCD	0.167	0.113	0.009	0.930
Overuse	−0.232	0.027	−0.389	<0.001
Harm	0.154	0.146	−0.427	<0.001
Potential Problems				
Necessity	0.203	0.054	−0.064	0.551
Concern	−0.226	0.031	0.061	0.567
NCD	0.333	<0.001	0.072	0.500
Overuse	−0.230	0.028	−0.269	0.011
Harm	−0.185	0.078	−0.305	0.004

South Asian patients were more satisfied with information about DMARDs if they perceived that they had a higher degree of personal control over symptoms (*P* < 0.001), that their treatment was more effective in controlling their symptoms (*P* = 0.014), and that they had a greater understanding of RA (*P* < 0.001). The White British patients who viewed their RA symptoms to be less fluctuant were more satisfied with the information received on potential problems (side effects of DMARDs) (Table 4). White British patients who reported more symptoms as associated with RA were less satisfied with information that they had received about the action and use of their DMARDs (*P* = 0.046). No other significant correlations were found between beliefs about medicines or patients’ satisfaction with information about RA treatment.

**Table 4:** Correlation between SIMS and IPQ domains within each ethnic group

	White British	*P* value	South Asian	*P* value
Action and usage				
Personal control	−0.015	0.887	0.540	<0.001
Treatment control	0.005	0.966	0.259	0.014
Illness coherence	0.091	0.389	0.469	<0.001
Potential Problems				
Personal control	0.083	0.431	0.446	<0.001
Treatment control	0.136	0.197	0.270	<0.001
Illness coherence	0.201	0.056	0.413	<0.001
Identity	−0.210	0.046	−0.074	0.493
Timeline cyclical	−0.289	0.005	0.037	0.732

Table 4 is only showing IPQ domains that were significant

### Medication adherence and ethnicity: univariable analysis

The distribution of the MARS scores by ethnicity is shown in Fig. 1. Using both the overall MARS score (*P* = 0.013, Mann–Whitney, as shown in Table 5) and treating it as a dichotomous variable (*P* = 0.011, Fisher’s exact test) there was a significant difference between the two ethnic groups, with 76.9 % of the White British group and 58.4 % of the South Asian group being high adherers.

**Table 5:** Univariable analysis of medication adherence (demographics and clinical data) (Data for all participants)

Categorical variables	Median MARS score (interquartile range)	*P* value
Gender		0.982*
M	28 (25–30)	
F	28 (24–30)	
Level of education		0.083**
Primary	26 (24–27)	
Secondary	27 (24–30)	
College	28 (25–30)	
University	28 (26–30)	
Employment		0.219**
Full time	28 (25–30)	
Part time	28 (22–30)	
Unemployment	30 (28–30)	
Never employed	23 (NA)	
Not working due to RA	26 (23–30)	
Not working for other reason	26 (22–30)	
Home maker	26 (24–29)	
Retired	29 (26–30)	
English spoken by patient		<0.001*
Yes	28 (26–30)	
No	24 (22–28)	
Same language spoken by patient and GP		0.002*
Yes	28 (25–30)	
No	24 (22–27)	
Patient Literacy level		0.053*
Yes	28 (24–30)	
No	24 (24–28)	
Ethnicity		0.013*
White British	28 (26–30)	
South Asian	26 (23–30)	
Oral /Biologic DMARDs		
Methotrexate		0.202*
Currently on	28 (24–30)	
Not on	26 (25–28)	
Sulphasalazine		0.058*
Currently on	26 (23–30)	
Not on	28 (25–30)	
Hydroxychloroquine		0.621*
Currently on	28 (22–30)	
Not on	28 (24–30)	
Anti-TNF		0.199*
Currently on	28 (24–30)	
Not on	27 (24–30)	
Patients’ country of birth		0.014**
UK	28^a^ (25–30)	
India	26^a^ (22–29)	
Pakistan	28 (27–30)	
Continuous variables (Spearman correlation)		
Age	0.093	0.212
Number of years of education	0.183^a^	0.014
Number of years in UK	−0.177	0.165
Disease duration	0.058	0.438
DAS CRP	0.032	0.674
IMD	−0.075	0.315

^a^Mann–Whitney, ^b^Kruskal Wallis ^*^ India vs UK P=0.0154 (Dunn’s Test) ^*^ = significant at <0.05, ^**^ = significant at <0.01. GP = General Practitioner

### Medication adherence and other variables: univariable analysis

There were significantly greater adherence scores in the English speaking patients (both South Asian and White British) (*P* < 0.001, Mann–Whitney) (Table 5). Patients who spoke with their GP in their preferred language had higher adherence scores (*P* = 0.002, Mann–Whitney). 71.2 % of those who spoke with GP using their preferred language were adherent compared with 35.3 % of those using another language. Patients who were born in the UK had higher adherence scores than those born in India (*P* = 0.015, Dunn’s test). Patients who had higher levels of education had higher adherence scores (*P* = 0.014, Spearman correlation). There was no significant difference between the DAS CRP scores for adherent and non-adherent patients (*P* = 0.94). The mean values were 3.89 and 3.90 respectively. The IMD score (deprivation level) was not associated with adherence scores for either group (White British, *P* = 0.320; South Asian, *P* = 0.503 Spearman correlation).

Patients who had higher General Overuse (*P* < 0.001, Spearman correlation) and General Harm scores (*P* < 0.001, Spearman correlation) had significantly lower adherence scores (Table 6). The NCD scores were correlated with MARS; patients whose perceived need for treatment outweighed their concerns about it had higher self-reported adherence scores (*P* = 0.005, Spearman correlation). SIMS components, action and usage (*P* < 0.001, Spearman correlation) and potential problems (*P* < 0.001, Spearman correlation), were correlated with MARS, with patients who were more satisfied with information on DMARDs having higher adherence scores. The two IPQ domains personal control (*P* = 0.012, Spearman correlation) and illness coherence (*P* < 0.001, Spearman correlation) were correlated with MARS, with patients who had better personal control and understanding of the disease having higher adherence scores.

**Table 6:** Univariable analysis of medication adherence (questionnaires) (Data for all participants)

Questionnaires		
BMQ		
Specific Necessity	0.052	0.489
Specific Concern	−0.114	0.127
NCD	0.209**	0.005
General Overuse	−0.309**	<0.001
General Harm	−0.300**	<0.001
SIMS		
SIMS action and usage	0.386**	<0.001
SIMS potential problems	0.469**	<0.001
HAQ	−0.055	0.465
IPQ		
IPQ Identity	−0.126	0.092
IPQ Timeline	0.071	0.343
IPQ Consequences	−0.052	0.492
IPQ Personal control	0.187*	0.012
IPQ Treatment control	0.085	0.258
IPQ Illness coherence	0.294**	<0.001
IPQ Timeline cyclical	−0.138	0.065
IPQ Emotional representation	−0.097	0.197

^*^ = significant at <0.05, ^**^ = significant at <0.01

### Multivariable analysis

Multivariable analysis was included first to look at age, sex and all variables significant in the univariable analysis. There were significant effects of ethnicity and both SIMS components on adherence scores and there was also a significant interaction between ethnicity and the SIMS action and usage component (Table 7). The effects were similar to those observed in the univariable analysis. South Asians had lower adherence scores compared to White British patients. Patients with higher SIMS scores had higher adherence scores. The interaction between SIMS action and usage and ethnicity was significant (*P* = 0.005, F test) with SIMS action and usage having a greater influence on adherence in South Asian than White British patients. We repeated the multivariable analysis using dichotomous MARS scores. The same variables were significant in both multivariable analyses with the exception that age was only significant when the MARS was treated as a binary variable. This suggested older patients were more likely to report lower adherence (*P* = 0.038).

**Table 7:** Multivariable analysis of medication adherence (Data for all participants)

	*B*	*Confidence interval*	*P value*	*R* ^*2* for model^
				0.328
Age (years)	−0.012	−0.049–(0.025)	0.523	
Gender (male)	−0.754	−1.754–(0.245)	0.138	
Number of years education	−0.022	−0.095–(0.050)	0.540	
English spoken by patient	−0.492	−2.384–(1.399)	0.608	
Same language spoken by patient and GP	1.447	−0.466–(3.360)	0.137	
Born in Pakistan^a^	2.071	−0.023–(4.165)	0.053	
Born in India^a^	1.575	−0.158–(3.307)	0.075	
Ethnicity (White British)	7.333	2.924–(11.743)	0.001*	
BMQ				
NCD	0.079	−0.590–(0.749)	0.815	
General Overuse	−0.193	−0.478–(0.092)	0.183	
General Harm	−0.049	−0.250–(0.153)	0.635	
SIMS				
SIMS action and usage (South Asian patients)	0.560	0.163–(0.958)	0.006*	
SIMS action and usage (White British patients)	−0.202	−0.595–(0.191)	0.311	
SIMS potential problems	0.428	0.217–(0.639)	<0.001**	
IPQ				
IPQ Personal control	−0.070	−0.205–(0.066)	0.310	
IPQ Illness coherence	0.086	−0.038–(0.211)	0.174	
Interaction				
SIMS action and usage x ethnicity^b^			0.005*	

^a^= reference category born in UK. ^b^The significant interaction indicates that the effect of SIMS action and usage varies with ethnic group: hence two separate sets of values for South Asian and White British patients. * = significant at <0.01 ** = significant at <0.001

## Discussion

This study is the first to show lower self-reported adherence amongst South Asian compared with White British RA patients. Lower self-reported adherence to DMARDs was associated with dissatisfaction with information about side effects (SIMS potential problems) in all patients. There was an interaction between ethnicity and dissatisfaction with information about both [1] potential problems associated with DMARDs (SIMS potential problems) and [2] how DMARDs work to control the condition (SIMS action and usage), such that dissatisfaction with information about the action and usage of and potential problems associated with DMARDs was a greater predictor of adherence scores in South Asian patients than in the White British patients. The adherence score was also associated with both specific and general beliefs about medicines. Patients who rated their concerns about DMARDS as high relative to their ratings of their personal need for DMARDs to control RA and maintain present and future health, reported lower self-reported adherence. DMARD adherence scores were also correlated with more negative views of medicines in general, with low adherence scores associated with a perception that medicines are fundamentally harmful and overused by doctors. The level of satisfaction with information correlated with negative beliefs about medicines and illness representation of their RA. South Asian patients had higher levels of satisfaction with information about DMARDs if they had a higher degree of personal control over symptoms.

Previous studies investigating the beliefs about medicines held by RA patients also found that patients had strong concerns about potential side effects of DMARDs [12, 19, 33]. Neame and Hammond [12] reported concern scores to be associated with non-adherence and our findings suggest that concerns about potential adverse consequences of DMARDs may be particularly prevalent in South Asian patients. This is consistent with our previous work where South Asian patients reported higher concerns about their DMARDs [14]. In another RA study, Treharne et al. [34] found that strong beliefs about the necessity of medications and believing medications not to be harmful predicted higher self-reported adherence but data were only available for White patients. In common with a previous study of medication beliefs and adherence in RA, we found no association, in our multivariable analysis, between self-reported adherence and sociodemographic factors (age, gender, level of education) or whether English was spoken by the patient. A study conducted in Bradford, UK, [35], suggested that the difficulty communicating in English experienced by some first-generation South Asian females was a barrier to understanding the disease process and the need for long term DMARD therapy. As a result, in that study, South Asian patients discontinued their DMARDs sooner than the non-South Asian population however, there were limited data on clinical outcomes. Although the DAS28 scores were slightly lower in the South Asian patients, it is difficult to draw significant conclusions related to disease severity based on a single DAS28 score. Longitudinal work is required to measure long term outcomes.

Our results suggest that dissatisfaction with information may occur because patients’ rationale for the long term use of DMARDs may not match with that of the health professional [17, 24, 36]. For example, the South Asian patients in our study who viewed RA to be a short lived condition may need to be provided with a convincing rationale for taking long-term treatment consistently even when their RA symptoms fluctuate. The association between beliefs and adherence seen within the current sample is consistent with theoretical predictions regarding the importance of beliefs about medicines in patients with RA [22]. These have been described in relation to the literature in other disease areas and the findings provide further support for the Necessity-Concerns framework in non-adherence, which suggests that these common-sense appraisals of treatment can impact on adherence [37]. Furthermore, Horne and Weinman [17] for example, reported that patients with asthma who were non-adherent had more doubts about the necessity of their medication and concerns about its adverse effects and believed asthma had a more negative impact on their life. Petrie et al. [38] demonstrated that it is possible to alter illness behaviours through targeting illness perceptions by providing a common-sense rationale for treatment and illness. Beliefs held by RA patients about treatment might arise from a range of factors including personal experience, illness representations and culture, as well as from information provided by healthcare professionals [39]. Furthermore, data in studies of RA [35], cardiovascular disease [40–42] and diabetes [43–47] in South Asian patients have cited cultural factors as an influence on patients’ response to illness and treatments. Work is now required in RA to build interventions that are based on theoretical models. For example, using the Necessity-Concerns framework and illness representations model at early stages of the diagnosis and initiation of treatment, may help to identify behaviours that influence decisions to take DMARDs and reduce non-adherence behaviours early.

Our study has a number of limitations including the fact that adherence was self-reported. We acknowledge that using a single approach to collect data on adherence is a limitation of this study. It is widely recognised that all individual approaches to measuring adherence have their specific limitations. For example, pharmacy refill data only index whether patients collect their medication, not whether they take it [9], while electronic monitoring methods can be expensive and inconvenient for patients to use (e.g. due to the size of pill caps [9]). The methodology to be used to measure adherence in the present study was discussed with our Patient Research Partners who recommended a self-report strategy [22]. Future studies in this population may need to combine a self-report measure with other techniques [48]. Secondly, this is the first study to have used a number of questionnaires (SIMS, IPQ and MARS) that were independently translated into three languages for the South Asian population. It is possible that some views specific to this population are not captured via these questionnaires. For example, in our previous qualitative work, patients’ views about disease, medicines and desired outcomes were influenced by their health beliefs [14, 49]. Furthermore, patients who responded to questionnaires via audio tapes could have missed the opportunity to record their own responses. Thirdly, this study was cross-sectional, preventing us from drawing conclusions regarding which factors were causally related to non-adherence especially the DAS scores. Fourthly, patients’ views about taking medications for other co-morbidities were not recorded; we acknowledge that this may have affected the views about taking DMARDs and would be an interesting issue to explore in future research. Finally, data were not collected on the delay from the time their RA began in patients commencing DMARDs; this could have been an important factor in explaining the different patterns of adherence as we have previously shown that RA patients of South Asian origin delay seeking medical help for longer than non-South Asians patients [15].

Despite these limitations, this study provides novel and useful insight into RA patients’ poor adherence in the two ethnic groups studied.

## Conclusions

In conclusion, non-adherence to DMARDs was associated with patients’ beliefs about DMARDs, their illness representations and views about medicines in general together with their satisfaction with the type and amount of information they had received about DMARDs. This varied between the two ethnic groups. Our data suggest the following recommendations for clinical practice; (1) Clinicians should engage with individual patients to identify specific factors that may be responsible for poor adherence behaviours. (2) Clinicians should ask patients about medication adherence during every consultation. (3) Clinicians should use tailored educational materials that provide an in-depth but comprehensible explanation of RA, the rationale for using DMARDs to control disease activity in RA and the consequences of poor adherence.

## Abbreviations

BMQ: beliefs about medicine
DMARD: disease modifying anti-rheumatic drug
HAQ: health assessment questionnaire
IPQ: illness perception questionnaire
MARS: medication adherence report scale
NCD: necessity concern differential
RA: rheumatoid arthritis
SIMS: satisfaction with information on medication scale
